# 4-(Furan-2-ylmeth­oxy)benzene-1,2-dicarbonitrile

**DOI:** 10.1107/S1600536811053669

**Published:** 2011-12-17

**Authors:** Hülya Tuncer, Ahmet Orhan Görgülü, Tuncer Hökelek

**Affiliations:** a Fırat University, Department of Chemistry, 23169 Elazığ, Turkey; b Hacettepe University, Department of Physics, 06800 Beytepe, Ankara, Turkey

## Abstract

In the title compound, C_13_H_8_N_2_O_2_, prepared from furfuryl alcohol and 4-nitro­phthalonitrile in the presence of potassium carbonate in dimethyl­formamide, the furan and benzene rings are oriented at a dihedral angle of 53.45 (9)°. In the crystal, weak C—H⋯O and C—H⋯N hydrogen bonds link the mol­ecules into a three-dimensional network.

## Related literature

For the use of phthalonitriles in the preparation of symmetrically and unsymmetrically substituted phthalocyanine complexes, see: Leznoff & Lever (1996 ▶). For the fundamental optical and electronic properties of phthalocyanines and their applications, see: McKeown (1998 ▶). For bond-length data, see: Allen *et al.* (1987 ▶).
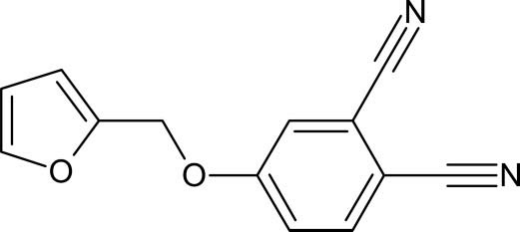


## Experimental

### 

#### Crystal data

C_13_H_8_N_2_O_2_
*M* *_r_* = 224.21
Orthorhombic, 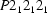

*a* = 3.9681 (2) Å
*b* = 14.3029 (3) Å
*c* = 19.2100 (5) Å
*V* = 1090.27 (7) Å^3^
*Z* = 4
Mo *K*α radiation
μ = 0.10 mm^−1^
*T* = 100 K
0.15 × 0.08 × 0.06 mm

#### Data collection

Bruker Kappa APEXII CCD area-detector diffractometer
Absorption correction: multi-scan (*SADABS*; Bruker, 2007 ▶) *T*_min_ = 0.986, *T*_max_ = 0.994
6208 measured reflections
2615 independent reflections
1709 reflections with *I* > 2σ(*I*)
*R*_int_ = 0.047

#### Refinement

*R*[*F* ^2^ > 2σ(*F* ^2^)] = 0.048
*wR*(*F* ^2^) = 0.141
*S* = 1.06
2615 reflections
154 parameters
H-atom parameters constrained
Δρ_max_ = 0.21 e Å^−3^
Δρ_min_ = −0.23 e Å^−3^

### 

Data collection: *APEX2* (Bruker, 2007 ▶); cell refinement: *SAINT* (Bruker, 2007 ▶); data reduction: *SAINT*; program(s) used to solve structure: *SHELXS97* (Sheldrick, 2008 ▶); program(s) used to refine structure: *SHELXL97* (Sheldrick, 2008 ▶); molecular graphics: *ORTEP-3 for Windows* (Farrugia, 1997 ▶); software used to prepare material for publication: *WinGX* (Farrugia, 1999 ▶) and *PLATON* (Spek, 2009 ▶).

## Supplementary Material

Crystal structure: contains datablock(s) I, global. DOI: 10.1107/S1600536811053669/bq2327sup1.cif

Structure factors: contains datablock(s) I. DOI: 10.1107/S1600536811053669/bq2327Isup2.hkl

Supplementary material file. DOI: 10.1107/S1600536811053669/bq2327Isup3.cml

Additional supplementary materials:  crystallographic information; 3D view; checkCIF report

## Figures and Tables

**Table 1:** Hydrogen-bond geometry (Å, °)

*D*—H⋯*A*	*D*—H	H⋯*A*	*D*⋯*A*	*D*—H⋯*A*
C7—H7⋯N1^i^	0.95	2.45	3.369 (4)	162
C10—H10⋯O2^ii^	0.95	2.42	3.233 (3)	144

Symmetry codes: (i) 
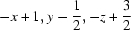
; (ii) 
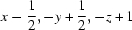
.

**Fig. 1.**
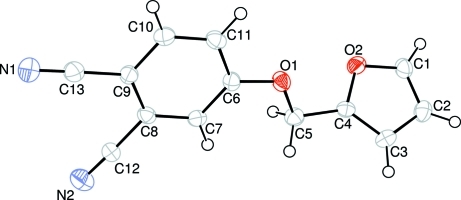
The molecular structure of the title molecule with the atom-numbering scheme. Displacement ellipsoids are drawn at the 50% probability level.

**Fig. 2.**
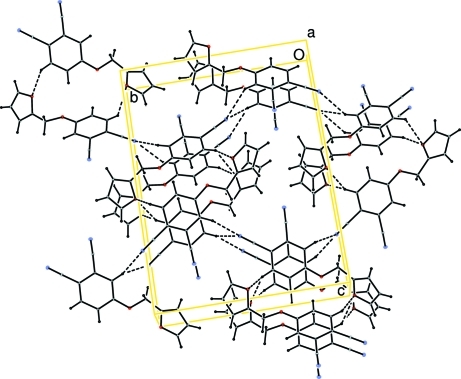
A partial packing diagram. Hydrogen bonds are shown as dashed lines.

## supplementary crystallographic information

### Comment

Phthalonitriles are used for preparing symmetrically and unsymmetrically substituted phthalocyanine complexes (Leznoff & Lever, 1996). Phthalocyanines have currently been the topic of research because of their wide application fields, such as thin film fabrication, organic pigments, chemical sensors, electrochromic display devices, molecular epitaxic deposition and composites, liquid crystals, photovoltaic cells self-assembled materials. The fundamental optical and electronic properties of these materials are explained and their potential in non-linear optics, optical data storage, electronic sensors, xerography, solar energy conversion, nuclear chemistry, molecular magnetism, electrochromic displays and heterogeneous catalysis is evaluated by McKeown (1998). The title compound was synthesized and its crystal structure is reported herein.

In the title compound, (Fig. 1), the bond lengths are close to standard values (Allen *et al.*, 1987). The furan [A (O2/C1—C4)] and the benzene [B (C6—C11)] rings are oriented at a dihedral angle of 53.45 (9)°. Atoms O1 and C5 are 1.094 (2) and -0.089 (3) Å away from the plane of ring A, while atoms O1, N1, N2, C5, C12 and C13 are -0.023 (2), -0.007 (3), 0.075 (3), 0.193 (3), 0.029 (3) and -0.003 (3) Å away from the plane of ring B, respectively. So, they are almost co-planar with the adjacent benzene ring.

In the crystal, weak intermolecular C—H···O and C—H···N hydrogen bonds (Table 1) link the molecules into a three-dimensional network (Fig. 2).

### Experimental

For the preparation of the title compound, furfuryl alcohol (1.49 g, 15.2 mmol) and 4-nitrophthalonitrile (2.64 g, 15.2 mmol) were heated at 358 K in dry DMF (15 ml) with stirring under argon atmosphere. Then, dry fine powdered potassium carbonate (6.00 g, 43.47 mmol) was added in portions (14 × 3.1 mmol) every 10 min. The mixture was heated for a further 24 h. After cooling, the mixture was added into ice-water (200 g). The product was filtered off and washed with NaOH solution (10%) and water until the filtrate was neutral. Recrystallization from ethanol gave a white product (yield: 1.25 g, 55.85%). Single crystals suitable for X-ray diffraction mesurement was obtained by slow evaporation of the solution in ethanol (m.p. 385-387 K).

### Refinement

The C-bound H-atoms were positioned geometrically with C—H = 0.95 Å and 0.99 Å, for aromatic and methylene H-atoms, respectively, and constrained to ride on their parent atoms, with *U*_iso_(H) = 1.2 *U*_eq_(C).

### Crystal data

**Table:**

C_13_H_8_N_2_O_2_	*F*(000) = 464
*M**_r_* = 224.21	*D*_x_ = 1.366 Mg m^−^^3^
Orthorhombic, *P*2_1_2_1_2_1_	Mo *K*α radiation, λ = 0.71073 Å
Hall symbol: P 2ac 2ab	Cell parameters from 1088 reflections
*a* = 3.9681 (2) Å	θ = 3.0–23.3°
*b* = 14.3029 (3) Å	µ = 0.10 mm^−^^1^
*c* = 19.2100 (5) Å	*T* = 100 K
*V* = 1090.27 (7) Å^3^	Rod-shaped, colorless
*Z* = 4	0.15 × 0.08 × 0.06 mm

### Data collection

**Table:**

Bruker Kappa APEXII CCD area-detector diffractometer	2615 independent reflections
Radiation source: fine-focus sealed tube	1709 reflections with *I* > 2σ(*I*)
graphite	*R*_int_ = 0.047
φ and ω scans	θ_max_ = 28.2°, θ_min_ = 2.1°
Absorption correction: multi-scan (*SADABS*; Bruker, 2007)	*h* = −4→5
*T*_min_ = 0.986, *T*_max_ = 0.994	*k* = −18→18
6208 measured reflections	*l* = −21→24

### Refinement

**Table:**

Refinement on *F*^2^	Primary atom site location: structure-invariant direct methods
Least-squares matrix: full	Secondary atom site location: difference Fourier map
*R*[*F*^2^ > 2σ(*F*^2^)] = 0.048	Hydrogen site location: inferred from neighbouring sites
*wR*(*F*^2^) = 0.141	H-atom parameters constrained
*S* = 1.06	*w* = 1/[σ^2^(*F*_o_^2^) + (0.0634*P*)^2^] where *P* = (*F*_o_^2^ + 2*F*_c_^2^)/3
2615 reflections	(Δ/σ)_max_ < 0.001
154 parameters	Δρ_max_ = 0.21 e Å^−^^3^
0 restraints	Δρ_min_ = −0.23 e Å^−^^3^

### Special details

**Table:**

Geometry. All esds (except the esd in the dihedral angle between two l.s. planes) are estimated using the full covariance matrix. The cell esds are taken into account individually in the estimation of esds in distances, angles and torsion angles; correlations between esds in cell parameters are only used when they are defined by crystal symmetry. An approximate (isotropic) treatment of cell esds is used for estimating esds involving l.s. planes.
Refinement. Refinement of F^2^ against ALL reflections. The weighted R-factor wR and goodness of fit S are based on F^2^, conventional R-factors R are based on F, with F set to zero for negative F^2^. The threshold expression of F^2^ > 2sigma(F^2^) is used only for calculating R-factors(gt) etc. and is not relevant to the choice of reflections for refinement. R-factors based on F^2^ are statistically about twice as large as those based on F, and R- factors based on ALL data will be even larger.

### Fractional atomic coordinates and isotropic or equivalent isotropic displacement parameters (Å^2^)

**Table:**

	*x*	*y*	*z*	*U*_iso_*/*U*_eq_	
O1	−0.0385 (5)	0.13224 (11)	0.56836 (9)	0.0273 (5)	
O2	0.0884 (5)	−0.01570 (12)	0.46812 (10)	0.0298 (5)	
N1	0.3842 (7)	0.51178 (17)	0.75462 (12)	0.0422 (7)	
N2	0.6574 (7)	0.26606 (17)	0.82143 (14)	0.0400 (7)	
C1	−0.0302 (8)	−0.09143 (17)	0.43144 (15)	0.0309 (7)	
H1	0.0059	−0.1016	0.3831	0.037*	
C2	−0.2035 (8)	−0.14879 (19)	0.47319 (15)	0.0311 (7)	
H2	−0.3107	−0.2056	0.4602	0.037*	
C3	−0.1961 (8)	−0.10832 (18)	0.54078 (15)	0.0303 (7)	
H3	−0.2976	−0.1328	0.5817	0.036*	
C4	−0.0165 (7)	−0.02822 (17)	0.53552 (14)	0.0236 (6)	
C5	0.0991 (8)	0.04146 (16)	0.58672 (14)	0.0269 (6)	
H5A	0.0227	0.0235	0.6339	0.032*	
H5B	0.3483	0.0443	0.5868	0.032*	
C6	0.0587 (7)	0.20592 (17)	0.60840 (14)	0.0230 (6)	
C7	0.2357 (7)	0.19683 (18)	0.67054 (14)	0.0236 (6)	
H7	0.2995	0.1369	0.6873	0.028*	
C8	0.3172 (7)	0.27691 (18)	0.70750 (14)	0.0234 (6)	
C9	0.2258 (7)	0.36548 (18)	0.68341 (13)	0.0239 (6)	
C10	0.0516 (7)	0.37317 (18)	0.62082 (13)	0.0264 (6)	
H10	−0.0107	0.4331	0.6037	0.032*	
C11	−0.0306 (7)	0.29413 (17)	0.58357 (14)	0.0262 (6)	
H11	−0.1487	0.2997	0.5408	0.031*	
C12	0.5042 (8)	0.26926 (18)	0.77133 (15)	0.0272 (7)	
C13	0.3132 (8)	0.4472 (2)	0.72251 (15)	0.0303 (7)	

### Atomic displacement parameters (Å^2^)

**Table:**

	*U*^11^	*U*^22^	*U*^33^	*U*^12^	*U*^13^	*U*^23^
O1	0.0331 (11)	0.0226 (9)	0.0264 (10)	0.0029 (9)	−0.0048 (9)	−0.0034 (8)
O2	0.0384 (12)	0.0241 (9)	0.0268 (10)	−0.0047 (9)	0.0026 (10)	−0.0003 (8)
N1	0.0552 (19)	0.0336 (13)	0.0378 (16)	−0.0074 (14)	−0.0024 (14)	−0.0029 (13)
N2	0.0422 (16)	0.0472 (16)	0.0304 (16)	0.0012 (13)	−0.0040 (14)	0.0020 (13)
C1	0.0389 (18)	0.0255 (14)	0.0282 (16)	−0.0005 (14)	−0.0043 (15)	−0.0053 (12)
C2	0.0318 (17)	0.0240 (14)	0.0374 (18)	−0.0012 (13)	−0.0028 (14)	−0.0039 (13)
C3	0.0314 (16)	0.0274 (14)	0.0321 (17)	−0.0040 (13)	0.0049 (14)	0.0055 (13)
C4	0.0241 (14)	0.0261 (13)	0.0206 (14)	0.0008 (13)	0.0029 (12)	0.0018 (11)
C5	0.0299 (16)	0.0225 (13)	0.0282 (15)	0.0041 (12)	−0.0014 (13)	0.0017 (12)
C6	0.0220 (14)	0.0243 (13)	0.0228 (14)	−0.0008 (12)	0.0024 (11)	−0.0032 (11)
C7	0.0236 (14)	0.0241 (13)	0.0231 (15)	−0.0008 (12)	0.0011 (12)	0.0041 (11)
C8	0.0221 (14)	0.0289 (15)	0.0194 (14)	−0.0014 (12)	0.0015 (12)	0.0010 (12)
C9	0.0258 (15)	0.0245 (13)	0.0216 (15)	−0.0023 (12)	0.0041 (12)	−0.0023 (12)
C10	0.0276 (16)	0.0250 (13)	0.0267 (15)	0.0026 (13)	0.0019 (13)	0.0030 (12)
C11	0.0227 (14)	0.0323 (14)	0.0237 (14)	0.0011 (13)	−0.0015 (13)	0.0028 (12)
C12	0.0299 (16)	0.0277 (15)	0.0239 (16)	−0.0015 (13)	−0.0016 (14)	−0.0007 (12)
C13	0.0358 (17)	0.0275 (15)	0.0277 (17)	0.0004 (14)	0.0001 (14)	0.0002 (13)

### Geometric parameters (Å, °)

**Table:**

O1—C5	1.452 (3)	C5—H5A	0.9900
O1—C6	1.360 (3)	C5—H5B	0.9900
O2—C1	1.375 (3)	C6—C7	1.391 (4)
O2—C4	1.372 (3)	C7—H7	0.9500
N1—C13	1.146 (3)	C8—C7	1.386 (4)
N2—C12	1.139 (4)	C8—C12	1.437 (4)
C1—H1	0.9500	C9—C8	1.397 (4)
C2—C1	1.338 (4)	C9—C10	1.391 (4)
C2—H2	0.9500	C10—C11	1.377 (3)
C3—C2	1.422 (4)	C10—H10	0.9500
C3—H3	0.9500	C11—C6	1.395 (3)
C4—C3	1.353 (4)	C11—H11	0.9500
C5—C4	1.473 (4)	C13—C9	1.432 (4)
			
C6—O1—C5	116.7 (2)	O1—C6—C7	123.8 (2)
C4—O2—C1	106.1 (2)	O1—C6—C11	115.8 (2)
O2—C1—H1	124.7	C7—C6—C11	120.4 (2)
C2—C1—O2	110.6 (2)	C6—C7—H7	120.6
C2—C1—H1	124.7	C8—C7—C6	118.7 (2)
C1—C2—C3	106.7 (2)	C8—C7—H7	120.6
C1—C2—H2	126.7	C7—C8—C9	121.3 (2)
C3—C2—H2	126.7	C7—C8—C12	119.6 (2)
C2—C3—H3	126.6	C9—C8—C12	119.1 (2)
C4—C3—C2	106.7 (3)	C8—C9—C13	120.2 (2)
C4—C3—H3	126.6	C10—C9—C8	119.1 (2)
O2—C4—C5	116.6 (2)	C10—C9—C13	120.6 (2)
C3—C4—O2	109.9 (2)	C9—C10—H10	119.9
C3—C4—C5	133.4 (2)	C11—C10—C9	120.1 (2)
O1—C5—C4	109.0 (2)	C11—C10—H10	119.9
O1—C5—H5A	109.9	C6—C11—H11	119.8
O1—C5—H5B	109.9	C10—C11—C6	120.3 (2)
C4—C5—H5A	109.9	C10—C11—H11	119.8
C4—C5—H5B	109.9	N2—C12—C8	177.7 (3)
H5A—C5—H5B	108.3	N1—C13—C9	179.0 (3)
			
C8—C9—C10—C11	0.4 (4)	C5—C4—C3—C2	−175.4 (3)
C13—C9—C10—C11	179.9 (3)	C4—C3—C2—C1	−0.1 (3)
C9—C10—C11—C6	0.3 (4)	C3—C2—C1—O2	−0.2 (3)
C5—O1—C6—C7	−10.1 (4)	C4—O2—C1—C2	0.4 (3)
C5—O1—C6—C11	170.0 (2)	C10—C9—C8—C7	−0.5 (4)
C10—C11—C6—O1	179.0 (3)	C13—C9—C8—C7	−179.9 (3)
C10—C11—C6—C7	−0.9 (4)	C10—C9—C8—C12	178.5 (3)
C6—O1—C5—C4	−176.6 (2)	C13—C9—C8—C12	−1.0 (4)
C1—O2—C4—C3	−0.4 (3)	C9—C8—C7—C6	−0.2 (4)
C1—O2—C4—C5	176.1 (2)	C12—C8—C7—C6	−179.1 (2)
O1—C5—C4—C3	−121.0 (3)	O1—C6—C7—C8	−179.1 (3)
O1—C5—C4—O2	63.5 (3)	C11—C6—C7—C8	0.8 (4)
O2—C4—C3—C2	0.3 (3)		

### Hydrogen-bond geometry (Å, °)

**Table:**

*D*—H···*A*	*D*—H	H···*A*	*D*···*A*	*D*—H···*A*
C7—H7···N1^i^	0.95	2.45	3.369 (4)	162
C10—H10···O2^ii^	0.95	2.42	3.233 (3)	144

Symmetry codes: (i) −*x*+1, *y*−1/2, −*z*+3/2; (ii) *x*−1/2, −*y*+1/2, −*z*+1.
